# Laboratory evaluation of the Chembio Dual Path Platform HIV-Syphilis Assay

**DOI:** 10.4102/ajlm.v5i1.433

**Published:** 2016-09-15

**Authors:** Mireille B. Kalou, Arnold Castro, Amy Watson, Heather Jost, Stacy Clay, Ye Tun, Cheng Chen, Kevin Karem, John N. Nkengasong, Ronald Ballard, Bharat Parekh

**Affiliations:** 1 International Laboratory Branch, Division of Global HIV and Tuberculosis, Center for Global Health, US Centers for Disease Control and Prevention, Atlanta, Georgia, United States; 2 Laboratory Reference and Research Branch, Division of STD Prevention, National Center for HIV/AIDS, Viral Hepatitis, STD and TB Prevention, US Centers for Disease Control and Prevention, Atlanta, Georgia, United States; 3 Office of the Associate Director of Laboratory Science, Center for Global Health, US Centers for Disease Control and Prevention, Atlanta, Georgia, United States

## Abstract

**Background:**

Use of rapid diagnostic tests for HIV and syphilis has increased remarkably in the last decade. As new rapid diagnostic tests become available, there is a continuous need to assess their performance and operational characteristics prior to use in clinical settings.

**Objectives:**

In this study, we evaluated the performance of the Chembio Dual Path Platform (DPP^®^) HIV–Syphilis Assay to accurately diagnose HIV, syphilis, and HIV/syphilis co-infection.

**Method:**

In 2013, 990 serum samples from the Georgia Public Health Laboratory in Atlanta, Georgia, United States were characterised for HIV and syphilis and used to evaluate the platform. HIV reference testing combined third-generation Enzyme Immunoassay and Western Blot, whereas reference testing for syphilis was conducted by the *Treponema pallidum* passive particle agglutination method and the TrepSure assay. We assessed the sensitivity and specificity of the DPP assay on this panel by comparing results with the HIV and syphilis reference testing algorithms.

**Results:**

For HIV, sensitivity was 99.8% and specificity was 98.4%; for syphilis, sensitivity was 98.8% and specificity was 99.4%. Of the 348 co-infected sera, 344 (98.9%) were detected accurately by the DPP assay, but 11 specimens had false-positive results (9 HIV and 2 syphilis) due to weak reactivity.

**Conclusion:**

In this evaluation, the Chembio DPP HIV–Syphilis Assay had high sensitivity and specificity for detecting both HIV and treponemal antibodies. Our results indicate that this assay could have a significant impact on the simultaneous screening of HIV and syphilis using a single test device for high-risk populations or pregnant women needing timely care and treatment.

## Introduction

Both syphilis and HIV infections can cause significant morbidity and mortality and are important public health concerns, especially in resource-limited settings (RLS). While the number of HIV-positive individuals continues to decline, in 2013, the Joint United Nations Program on HIV/AIDS (UNAIDS) estimated that 32.6 million people were still living with HIV in low- and middle income countries.^1^ Similarly, approximately 90% of new syphilis cases globally occur in low-income countries where sexually-transmitted HIV is also a major public health problem.^2^ Syphilis is common among individuals with HIV, and the risk of acquiring HIV is estimated to increase exponentially when syphilis is present.^3,4^ The World Health Organization (WHO) has estimated that more than 12 million new cases of adult syphilis occur worldwide each year, and the disease can be transmitted congenitally, affecting 500 000 or more infants annually.^5^ Among pregnant women, the transmission of HIV and syphilis infections to their unborn infants can result in serious adverse pregnancy outcomes, such as premature delivery, low birth weight, congenital anomalies and perinatal death.^6,7,8^

WHO and UNICEF guidelines on essential maternal and child health services provide recommendations that all pregnant women have a laboratory profile including testing for HIV and syphilis.^9^ However, in RLS, most women receive their maternal and child health services at the lowest level of the tiered health systems, with very limited laboratory capacity.^10^ Over the past decade, the development of single infectious disease rapid diagnostics has allowed detection and treatment to take place on-site, even in low-level health facilities that lack basic public laboratory infrastructure.^9,10^ Unfortunately, sexually-transmitted infections (STIs) are often not viewed as a public health priority in many RLS. STI surveillance, prevention and treatment programmes are generally poorly resourced and staffed. However, as technology advances, a single device capable of screening multiple diseases could increase the uptake of syphilis testing, especially in RLS, where syphilis infection often remains undiagnosed because routine testing is not part of the national guidelines.

As global efforts continue to scale-up programmes for screening, treatment and prevention of both HIV and syphilis, the use of accurate rapid diagnostic tests (RDTs) remains a reliable and cost-effective tool for RLS.^8,11,12^ RDTs allow access to testing in geographic areas where laboratory services are limited and can be performed by staff with minimal training, such as antenatal care settings, tuberculosis clinics, and clinics serving hard-to-reach populations.^13,14,15,16,17^ The use of RDTs also decreases the turnaround time and the overall cost of the testing, which could contribute significantly to the uptake of testing and the acceptance by countries with limited resources.

The availability of RDTs designed to screen individual infectious diseases, including HIV and syphilis, has increased remarkably in the past decade.^11,12,18,19^ Recently, the multiplexing of RDTs has also been developed to address operational challenges around confirmatory testing, turnaround time and specimen volume required to perform multiple tests.^20,21,22^ With the introduction of integrated approaches, such as the Making Pregnancy Safer Initiative and dual elimination of mother-to-child transmission of HIV and syphilis, more national programmes are advocating for combined screening and treatment of these two diseases.^23^ To support these programmes, a single RDT device to screen simultaneously for both HIV and syphilis using finger-prick blood is vital in order to be cost-effective, increase coverage and manage supply chain challenges.^24^ Therefore, the availability of a quality dual rapid test for HIV and syphilis would greatly strengthen the prevention and control programmes that target the most at-risk populations and mother-to-child transmission of HIV and syphilis.

The aim of this study was to evaluate the performance characteristics of the Chembio Dual Path Platform (DPP)^®^ HIV–Syphilis Assay (Chembio, Medford, New York, United States; hereafter termed DPP HIV–Syphilis Assay) and to determine its eligibility for inclusion in the US Agency for International Development procurement waiver list for RDTs intended for use in countries supported by the United States President’s Emergency Plan for AIDS Relief (PEPFAR).

## Methods

### Ethical considerations

This study was conducted under an existing protocol which was submitted for human subjects review and approval at the US Centers for Disease Control and Prevention (CDC). The specimens used in this evaluation were obtained as part of an existing Material Transfer Agreement with the Laboratory Reference and Research Branch, Division of STD Prevention, CDC. Consent was sought according to the Georgia Public Health Laboratory policy.

### Specimen characterisation and reference testing algorithm

In 2013, we prospectively collected 1006 sera from the Georgia Public Health Laboratory in Atlanta, Georgia, United States. The specimens, normally discarded, were delinked from personal identifiers or any other demographic information (i.e., age, gender, etc.) and unique CDC identifiers were assigned. Specimens were stored at −70 °C until ready for testing.

These sera were characterised and constituted the evaluation panel. The serum panel was initially characterised for syphilis by qualitative *Treponema pallidum* passive particle agglutination (TPPA; Fujirebio Diagnostics, Inc., Malvern, Pennsylvania, United States), after which all TPPA-positive specimens were confirmed by TrepSure (Trinity Biotech, Jamestown, New York, United States) testing. Additionally, all 1006 specimens were screened for HIV using a US FDA-approved Enzyme Immunoassay (EIA; Genetic systems HIV-1/HIV-2 Plus O EIA, Bio-Rad, Hercules, California, United States). All reactive specimens by the EIA were further confirmed by a US FDA-approved Western Blot (WB) assay (Cambridge Biotech HIV-1 Western Blot, Cambridge Biotech Corporation, Rockville, Maryland, United States). All specimens with incomplete HIV and/or syphilis test results, including specimens with HIV WB indeterminate results, were excluded from the evaluation.

### Chembio DPP HIV–Syphilis Assay performance characteristics

The DPP HIV–Syphilis Assay is a single-use immune-chromatographic rapid screening test for the detection of specific antibodies against HIV types 1 and 2 (HIV 1/2) and *T. pallidum*, with either finger-stick whole blood, venous whole blood, serum, or plasma samples. Two trained operators independently performed and interpreted the assay according to the manufacturer’s instructions, and the test results were recorded on separate sheets. Any visible band in the positive region was considered as a positive result for HIV and/or syphilis, irrespective of the strength of the band. Performance characteristics of the DPP HIV–Syphilis Assay were determined by comparing the assay with the HIV and syphilis gold standards as outlined above. The eligibility criteria for inclusion of Chembio DPP HIV-Syphilis Assay in the USAID waiver procurement list of RDTs were sensitivity > 99% and specificity > 98% for the HIV line and sensitivity 94% and specificity > 95% for syphilis line.

### Inter-reader and inter-lot variability assessment

To assess the consistency of the assay’s performance, three different test lots were evaluated using dilution panels totaling 100 individual specimens for each biomarker. The panels were created from a five-fold serial dilution using 10 HIV-positive and 10 syphilis-positive sera with strong reactivity prepared in pooled negative plasma (SeraCare Life Science, Gaithersburg, Maryland, United States). The end-point detection limits of each lot were determined by comparing the performance of the reference lot (Lot #1) against Lot #2 and Lot #3. The reference lot was used to establish the sensitivities and specificities of the assay. The performance of each lot was considered acceptable, if the lot was within at least one dilution end-point titre from the reference lot, with an overall agreement of ≥ 90%.^25^ If a lot differed by > 10% from the reference lot then it was considered unacceptable.

Due to the subjectivity in interpretation of the RDT results, we also assessed inter-reader variability of the assay by independently comparing the results interpreted and recorded by a total of three technicians, including the two technicians who performed the testing.

### Assessment of operational characteristics

The rapid scale-up of most HIV and syphilis testing programmes involves the use of RDTs, most often performed by health professionals with a wide range of expertise in a variety of settings. Therefore, it is crucial to assess key operational characteristics, such as the ease of use, number of steps, storage conditions and ease of interpretation, which might impact the large-scale implementation of the DPP HIV-Syphilis Assay in RLS.

For quality assurance purposes, two different technicians performed testing of 10 test devices at a time, to allow for sufficient reading time. Each day, a set of positive and negative controls for HIV and syphilis was run by each technician prior to testing the evaluation specimens. The test results were recorded on a worksheet by the technician performing testing and verified by a second technician.

### Data analysis

All test results were entered independently by two technicians into a Microsoft Excel spreadsheet (Microsoft Corporation, Redmond, Washington, United States). The spreadsheets were reviewed for accuracy and merged prior to data analysis. The analysis included the calculation of the performance characteristics of the DPP HIV–Syphilis Assay compared to the HIV and syphilis predicate testing results (i.e., sensitivities, specificities, their corresponding 95% confidence intervals [CIs] and kappa values). The kappa values were computed using Stata Software (StataCorp LP, College Station, Texas, United States). We also determined the ability of the DPP HIV–Syphilis Assay to accurately identify HIV only, syphilis only, and co-infected (both HIV- and syphilis-positive) specimens.

## Results

### Panel characterisation data

Of the 1006 serum samples tested by the reference algorithms (EIA/WB for HIV and TPPA/TrepSure for syphilis), 16 (1.6%) specimens with incomplete/invalid predicate HIV and/or syphilis testing results were excluded from the evaluation. Thus, 990 (98.4%) fully characterised sera for both biomarkers were included in the final analysis. Of the 990 sera, 79 (7.9%) were HIV-positive, 299 (30.2%) were syphilis-positive, 348 (35.2%) were both HIV- and syphilis-positive, and 264 (26.7%) were negative for both syphilis and HIV (Table 1).

**TABLE 1:** Composition of the evaluation panel characterised for HIV and syphilis in 2013 (*N* = 990).

Syphilis reference testing	HIV reference testing†		
	Positive (%)	Negative (%)	Total (%)
Positive (%)	348 (35.2)	299 (30.2)	647 (65.4)
Negative (%)	79 (7.9)	264 (26.7)	343 (34.6)
**Total**	**427 (43.1)**	**563 (56.9)**	**990 (100.0)**

† Composition of the evaluation panel obtained from the Georgia State Health Department characterised by the US FDA-approved EIA and Western blot for HIV and TPPA and TrepSure for syphilis screening.

### Performance characteristics of the DPP HIV-Syphilis Assay

Of the 427 samples confirmed to be HIV-positive by the HIV EIA/WB reference algorithm, only one sample was identified as HIV-negative by the DPP HIV–Syphilis Assay, resulting in a sensitivity of 99.8% (95% CI: 98.7% – 100%) (Table 2). Similarly, of the 563 samples confirmed to be HIV-negative by the HIV reference algorithm, 554 samples were identified as HIV-negative by the DPP HIV–Syphilis Assay. Thus, the specificity of the assay was 98.4% (95% CI: 97.0% – 99.3%) for the HIV component.

**TABLE 2:** Performance characteristics of the Chembio DPP HIV-Syphilis Assay when compared to HIV and syphilis reference testing algorithms (*N* = 990).

Result	True positive	False negative	True negative	False positive	Sensitivity [95% CI]	Specificity [95% CI]	Kappa-value
HIV line	426	1	554	9	99.8 [98.7% – 100%]	98.4 [97.0% – 99.3%]	0.98
Syphilis line	639	8	341	2	98.8 [97.6% – 99.5%]	99.4 [97.9% – 99.9%]	0.98

CI, confidence interval.

Of the 647 samples confirmed to be syphilis-positive by the TPPA/TrepSure reference algorithm, 639 were identified as syphilis-positive by the DPP HIV-Syphilis Assay, resulting in a sensitivity of 98.8% (95% CI: 97.6% – 99.5%). Only two of the samples confirmed to be syphilis-negative by the syphilis reference algorithm were identified as positive by the DPP HIV-Syphilis Assay, resulting in a specificity of 99.4% (95% CI: 97.9% – 99.9%) (Table 2).

Of the 990 sera, 20 (2.0%) were discordant between the DPP HIV–Syphilis Assay and the predicate testing for both HIV and syphilis. These 20 specimens included 11 false positives (9 HIV and 2 syphilis) and nine false negatives (1 HIV and 8 syphilis). The 11 false positives were repeated using the DPP HIV-Syphilis Assay and the results did not change. The kappa-values indicated high agreement between the DPP HIV-Syphilis Assay and the reference testing algorithms (kappa-value: 0.98 for both methods).

A comparison of the DPP HIV–Syphilis Assay results with the reference testing data showed that 344 (98.9%) of the 348 dually-reactive samples were confirmed by the reference methods (Table 3). However, the remaining four specimens (1.1%) identified as dually-reactive by the reference testing were identified as positive for syphilis by the DPP HIV–Syphilis Assay, but negative for HIV. No invalid results were observed during the evaluation.

**TABLE 3:** Comparison of Chembio DPP HIV–Syphilis Assay results and HIV and syphilis reference testing (*N* = 990).

DPP HIV–Syphilis testing	HIV and syphilis reference testing				
	Syphilis (%)	HIV (%)	Co-infection (%)	Negative (%)	Total (%)
Syphilis (%)	294 (29.7)	0 (0.0)	0 (0.0)	1 (0.1)	**295 (27.8)**
HIV (%)	0 (0.0)	77 (7.8)	4 (0.4)	8 (0.8)	**89 (8.9)**
Co-infection (%)	1 (0.1)	1 (0.1)	344 (34.7)	0 (0.0)	**346 (34.9)**
Negative (%)	4 (0.4)	1 (0.1)	0 (0.0)	255 (25.6)	**260 (26.3)**
**Total**	**299 (30.2)**	**79 (7.9)**	**348 (35.2)**	**264 (26.7)**	**990 (100)**

### Inter-lot and inter-operator variability

Compared to the reference lot, the performance of Lot #2 differed by 3% for HIV and 6% for syphilis, whereas Lot #3 differed by 4% for HIV and 3% for syphilis. In addition, there was high consistency in the interpretation of the DPP HIV–Syphilis Assay results for both HIV (96%) and syphilis (91%) among three different technicians. Both inter-lot and inter-operator variability were considered acceptable because both were less than 10%.

### Operational characteristics of the DPP HIV-Syphilis Assay

The test device included two ports and two buffer vials, which could potentially lead to confusion with the pre-dilution step of the blood in buffer and possible mix-ups at the additional steps. The product evaluated also did not include a specimen application device (i.e., disposable micropipette). Some level of complexity in the interpretation of the results of this three-line test required appropriate training. However, the run time (15–20 minutes per sample), long shelf life and individual packaging added value to the high performance of the assay.

## Discussion

The DPP HIV-Syphilis Assay displayed high sensitivity and specificity for detecting HIV and syphilis in co-infected individuals and met the minimum requirements for inclusion in the USAID procurement waiver list, thus making it accessible to PEPFAR supportive countries that may consider including it to their current national HIV testing algorithms. The high agreement between the DPP HIV-Syphilis Assay and the reference testing algorithms demonstrates the ability of the dual rapid test to accurately identify individuals co-infected with syphilis and HIV. While previous studies have suggested that the interpretation of serological assays for syphilis can be challenging in HIV-positive patients,^26^ our evaluation demonstrates that the DPP HIV-Syphilis Assay can accurately detect syphilis in HIV-positive individuals.

We observed eight false-negative syphilis results with the DPP HIV-Syphilis Assay. These findings are comparable with previous studies which have found that, while uncommon, false-negative syphilis results may occur among HIV-positive individuals when using serological tests such as the quantitative rapid plasma reagin test and the TPPA assay, especially during the late stages of the disease.^27^ Similarly, the nine false-positive HIV results identified by the DPP HIV-Syphilis Assay could be attributed to serological cross-reactivity or non-specific immune reactivity, sometimes observed with HIV rapid tests.^28^ Reassuringly, there were only two false-positive syphilis results identified by the DPP HIV-Syphilis Assay, demonstrating the high specificity of the test for syphilis detection, as has been reported previously.^20^

With the massive roll out of RDTs in most RLS, the need for a laboratory-based confirmatory test for HIV and syphilis is no longer required to initiate treatment. The recently-released WHO guidelines on HIV Testing Services recommends that countries and programmes implement the retesting strategy for verification purposes.^29^ As countries adopt these recommendations, all patients with an initial HIV-positive result will be retested by a different healthcare worker at the treatment centre prior to initiating care and treatment, minimizing the turnaround time for returning results to the referring clinic which would occur if the confirmatory testing was conducted in a laboratory setting.^30,31^ Although there has been concern that healthcare providers who typically work alone in settings offering integrated services may be unable to efficiently perform several individual rapid diagnostics within a single visit, it is important to highlight that with the increased focus on task-sharing strategies to address staffing issues, healthcare professionals routinely perform multi-test algorithms to screen HIV patients, in addition to other duties. Thus, the inclusion of a multi-disease single test into the current HIV multi-test algorithm may help decrease work load and increase the uptake of syphilis testing.^32^

Screening multiple diseases with a single test device such as the DPP HIV-Syphilis Assay provides the opportunity to potentially strengthen health systems. The main operational considerations should be training on the different RTDs as they are introduced, and robust monitoring of providers’ performance through the implementation of quality assurance measures, such as use of standard operating procedures or job aides, proficiency testing panels and proper documentation of the test results in a standardised logbook or register that can be reviewed to identify issues and provide corrective actions.

The multiplex RDTs might help address issues observed in vertical programmes related to the cost of testing, quality assurance, and the integration of training to providers on use of the tests.^33^ While this evaluation did not include a cost analysis to determine the cost-effectiveness of the DPP HIV-Syphilis Assay, the current unit price, estimated at US $1.20 – $1.50, is comparable to that of other rapid tests commonly used by national HIV programmes and could be substituted for some of the screening tests combined in more cost-effective algorithms for both HIV and syphilis diagnosis.^24^ Moreover, in settings where test stock outs are recurrent because of the lack of proper supply chain management systems, the DPP HIV-Syphilis Assay may be a suitable alternative.

The DPP HIV-Syphilis Assay has the potential to screen for HIV and syphilis infections with a single test device in settings such as antenatal care and sexually-transmitted disease clinics in RLS where perinatal HIV and congenital syphilis are significant contributors to morbidity and mortality. The presence of STIs such as syphilis increases the risk of transmission of HIV.^34^ Failure to diagnose and immediately treat or provide appropriate care for a pregnant woman, her partner, and the infant may result in serious complications, ranging from foetal wastage, neonatal and infant infections, and premature death.^35^ In addition, the DPP HIV-Syphilis Assay may have applications both domestically and in international settings with limited on-site laboratory capacity and/or where loss to follow-up is high. Moreover, in settings where access to HIV testing is prohibitive due to stigma, a combined diagnostics method could lessen stigmatisation and increase access to testing.^28,29^

### Recommendations

Operationally, the DPP HIV-Syphilis Assay, considered to be a rapid test, requires a pre-dilution step, use of second buffer and multiple steps, which may add some level of complexity for providers with limited laboratory expertise. Moreover, the presence of three lines, one for control, a second for syphilis and a third for HIV, can lead to misinterpretation of results by less-trained individuals. Therefore, it would be important to ensure adequate training is provided prior to its use in the field. Appropriate labeling, for example colour coding of sample and buffer ports and matching buffer bottles, and careful interpretation of results with clear job aides to avoid mix-ups, may assist in ensuring that no errors are made. In addition, the use of standardised registers to monitor ongoing agreement between tests, enrollment of testers in an external quality assessment programme and close supervision and monitoring, are strongly recommended prior to routine roll out.

Similar to recent publications on dual HIV-syphilis RDTs, this study was a laboratory-based evaluation using a panel of well-characterised and archived serum samples.^36^ In the same study, all three dual HIV-syphilis RDTs exhibited high sensitivity and specify when performed in a laboratory setting and by trained personnel.

### Limitations

This evaluation did not determine the performance of RDTs outside the laboratory, by staff with limited training, or using fresh specimens obtained by fingerpick. As such, additional field evaluations will be needed. The findings of this evaluation were primarily based on a serum panel obtained from the local Georgia Public Health Laboratory, which may not necessarily be representative of samples from other geographic locations with varied rates of HIV/syphilis infections and antibody profiles. Nor does our study sample represent populations from countries with limited resources who will likely be tested by RDTs. Thus, there is a need for further field evaluations prior to the broader use of the DPP HIV-Syphilis Assay in clinical settings.

### Conclusion

This laboratory evaluation suggests that the Chembio DPP^®^ HIV–Syphilis Assay could be a suitable screening method for HIV and syphilis using the same device. This test was deemed eligible for inclusion in the USAID procurement waiver list for RDTs intended for use in PEPFAR supported countries. Moreover, it could improve the acceptability and increase the uptake of testing and treatment to accelerate elimination of mother-to-child transmission of syphilis and HIV. In addition, for high-risk populations, it could potentially increase uptake of testing, linkage to early care and treatment, and play an important role in syphilis control. In most instances, RDTs are reliable for screening HIV and syphilis; however, it is important to remember that misclassifications due to cross-reactivity or atypical immune response may occur. With the adoption of a task-sharing approach to address staff shortages in RLS, it will be important to develop a simple and practical job aides and emphasise hands-on training of healthcare providers in order to ensure that manufacturer testing procedures and national testing guidelines are followed so as to minimise operational errors. Moreover, further field evaluations should be conducted to assess the feasibility and acceptability of the DPP HIV–Syphilis Assay among healthcare workers and to determine its cost-effectiveness when included in the routine HIV testing algorithms.
